# Mono and Dual Cofactor Dependence of Human Cystathionine β-Synthase Enzyme Variants *In Vivo* and *In Vitro*

**DOI:** 10.1534/g3.113.006916

**Published:** 2013-10-01

**Authors:** Dago Dimster-Denk, Katherine W. Tripp, Nicholas J. Marini, Susan Marqusee, Jasper Rine

**Affiliations:** The California Institute for Quantitative Biosciences, Department of Molecular and Cellular Biology, University of California, Berkeley, California 94720

**Keywords:** cystathionine β-synthase, *Saccharomyces*, *CYS4*, vitamin B6, cysteine biosynthesis, heme

## Abstract

Any two individuals differ from each other by an average of 3 million single-nucleotide polymorphisms. Some polymorphisms have a functional impact on cofactor-using enzymes and therefore represent points of possible therapeutic intervention through elevated-cofactor remediation. Because most known disease-causing mutations affect protein stability, we evaluated how the *in vivo* impact caused by single amino acid substitutions in a prototypical enzyme of this type compared with physical characteristics of the variant enzymes *in vitro*. We focused on cystathionine β-synthase (CBS) because of its clinical relevance in homocysteine metabolism and because some variants of the enzyme are clinically responsive to increased levels of its B6 cofactor. Single amino-acid substitutions throughout the CBS protein caused reduced function *in vivo*, and a subset of these altered sensitivity to limiting B6-cofactor. Some of these B6-sensitive substitutions also had altered sensitivity to limiting heme, another CBS cofactor. Limiting heme resulted in reduced incorporation of heme into these variants, and subsequently increased protease sensitivity of the enzyme *in vitro*. We hypothesize that these alleles caused a modest, yet significant, destabilization of the native state of the protein, and that the functional impact of the amino acid substitutions caused by these alleles can be influenced by cofactor(s) even when the affected amino acid is distant from the cofactor binding site.

The interpretation of human genome variation is a defining challenge in human biology for the foreseeable future. The unprecedented increase in both total sequence information and in the number of new gene variants has created a critical bottleneck in the ability to interpret the impact of the genetic variants in an individual. Clearly, advances in interpretation are crucial to realize the potential of personalized medicine. As one measure of the magnitude of the challenge, any two unrelated humans differ by an average of 3 million single-nucleotide polymorphisms (SNPs). Among the SNPs in an individual, a current estimate is that 10,000 cause a change in the sequence of a protein (Abecasis *et al.* 2012; Tennessen *et al.* 2012). Moreover, each individual has changes in genes that have been implicated in human disease (Levy *et al.* 2007). The pace of SNP discovery far outpaces the speed at which polymorphisms are experimentally evaluated. For mutations that cause disease with Mendelian inheritance due to a single amino acid substitution, the vast majority is believed to decrease the stability (free energy of folding) of the protein, leading to partial or global unfolding and either aggregation or degradation (Yue *et al.* 2005). If this observation were to apply broadly to nonsynonymous SNPs, then robustly predictive calculations of the energetic effects caused by these mutations could, in principle, make a valuable contribution to genome sequence interpretation. Well-characterized sets of variant forms of multiple proteins are needed to help drive the development of such methods.

A particular subclass of sequence variants of great interest and potential importance is the set of mutations whose deleterious effects on proteins are readily remediable by simple means. The prototype for such mutations were first highlighted in bacterial genetic studies of mutations in genes encoding certain vitamin-dependent enzymes that can be suppressed by increased levels of their cognate vitamins (Guirard *et al.* 1971; Ames *et al.* 2002). In addition, some human mutations cause clinical phenotypes sensitive to remediation by increased vitamin dosages. We hypothesize that vitamin remediation occurs when a variant enzyme that has lost a crucial amount of free energy of folding can be compensated by the free energy of binding with the vitamin. In this case, the vitamin acts as a chemical splint, with the ligand-binding energy shifting the folding equilibrium and thereby making up for the partial loss of free energy caused by the mutation. Such variant proteins could be either dysfunctional, marginally functional, or substantially functional depending upon cofactor availability.

In the human MTHFR gene, the majority of nonsynonymous changes in this enzyme’s catalytic domain, found in a survey of nonclinical samples, have deleterious effects on the enzyme (Marini *et al.* 2008). Moreover, for this enzyme, which participates in folate-driven, one-carbon metabolism, the deleterious impact of most such genetic substitutions can be suppressed by simply increasing the level of folate available to the cell. There is no crystal structure available for human MTHFR, thereby precluding structure-based approaches to assess the impact of these mutations, although phylogenetic approaches are promising (Marini *et al.* 2010).

In the present study we turned to human cystathionine β-synthase (CBS), a vitamin-dependent enzyme whose structure is known, to explore the concept of cofactor remediation more deeply to determine its prevalence, and whether there are structural principles that can be illuminated with such alleles. In addition, well-characterized sets of alleles affecting a protein can serve as a test-bed for efforts such as the Critical Assessment of Genome Information (https://genomeinterpretation.org).

CBS catalyzes the first step of cysteine biosynthesis via the *trans*-sulfuration pathway, which consumes homocysteine produced from methionine metabolism. Human CBS requires two cofactors for function: vitamin B6 and heme. There are several biological forms of vitamin B6. The soluble form in vitamin supplements is pyridoxine, whereas the active form directly involved in CBS function is pyridoxal 5′-phospate (PLP). Homocystinuria due to CBS deficiency (OMIM 236200) is a recessive inborn-error of sulfur-amino acid metabolism, resulting in increased levels of homocysteine in the urine. More than 140 different disease-associated mutations have been identified in the CBS gene (Kraus *et al.* 1999, 2013). Several alleles encode proteins that are clearly pyridoxine remediable: A114V (pyridoxine Km variant), R266K, R369H, K384E, L539S, and the common I278T variant.

The choice of a B6-dependent enzyme allowed us to test the generality of the observations from our studies of MTHFR variants and their responses to folate supplementation. The rationale for the selection of CBS was several-fold: (1) an *in vivo* assay for CBS activity and vitamin-responsiveness is established (Kim *et al.* 1997; Kruger and Cox 1994, 1995; Mayfield *et al.* 2012); (2) the literature on CBS mutations and disease establish this enzyme as metabolically significant (Meier *et al.* 2003); (3) clinically relevant vitamin B6-responsive variants provide a benchmark for validation; and (4) the crystal structure allows for structural-based computational predictions of functional impact, including those based upon calculated free-energy-of folding changes (Meier *et al.* 2001). Clinically associated variants are inherently biased toward dysfunction. Therefore, for this study we focused our analysis on a set of designed variants with differing cofactor responses and examined possible conformational changes by *in vitro* measurements of thermolysin sensitivity, as recently demonstrated for studies of CBS (Hnizda *et al.* 2012a, 2010).

## Materials and Methods

### Plasmids

The plasmid pHUCBS was the kind gift of Warren Kruger (Kruger and Cox 1995). This plasmid contains the human CBS cDNA (mRNA reference sequence NM_000071, protein reference sequence NP_000062) and served as the source of the CBS coding region for all subsequent plasmid constructions. Polymerase chain reaction was used to amplify the CBS coding region and subclone the fragment into both a bacterial expression vector (2C-T, see *Protein expression and purification* below) as well as a yeast expression vector containing the *TEF1* promoter and *CYC1* terminator (p416-TEF; Mumberg *et al.* 1995). A derivative of this plasmid placed the hemagglutinin A epitope tag at the 3′-end of the CBS coding region (pJR2858). Site-directed CBS variants were constructed using the QuickChange II Kit from Agilent (Santa Clara, CA). Random variant libraries were created using the Diversity PCR Random Mutagenesis Kit from Clontech (Mountain View, CA) and cloned into yeast expression vectors by cotransformation with gapped vector directly into yeast, allowing homologous recombination to construct the expression plasmids. Plasmids were rescued from yeast into *Escherichia coli* and sequenced. All mutations were constructed in the CBS clone containing the hemagglutinin A epitope tag.

For all variant constructs, the entire CBS coding region was sequenced to ensure that a single amino acid change had been created. Each sequence-confirmed variant allele was then retransformed into yeast, and these transformants used for all subsequent analyses of phenotype and *in vivo* properties.

### Strains

A haploid *Saccharomyces cerevisiae* strain containing a complete deletion of the gene *CYS4*, encoding yeast cystathionine β-synthase, was obtained from the yeast knockout collection (Invitrogen), and served as the parent for all yeast strains used in this study (*MATα cys4*::*KanMX his3Δ1 leu2Δ0 lys2Δ0 ura3Δ0*). A *hem1Δ* strain, deficient in δ-aminolevulinate synthase, was created by the direct transformation of the *cys4Δ* parent with a *hem1*::*LEU2* construct. Expression plasmids carrying the human CBS major allele, or variants, were transformed into this strain by selecting for uracil prototrophy (Ito *et al.* 1983).

### Growth conditions

We adopted the strategy of Kruger *et al.* for the implementation of an *in vivo* CBS assay (Kim *et al.* 1997; Kruger and Cox 1994, 1995) and was essentially as described previously (Mayfield *et al.* 2012). In summary, plasmid-bearing strains were maintained on synthetic complete medium lacking uracil and containing glutathione (GSH), a tripeptide used as a source of cysteine to alleviate the need for functional CBS. Human CBS function was assayed based upon the growth of *cys4Δ* yeast strains in liquid and on solid minimal medium containing Yeast Nitrogen Base without vitamins (Qbiogene, Calsbad, CA) or GSH. Although these strains had wild-type versions of all *MET* genes, growth in minimal media also required the addition of methionine, presumably to augment a limiting intracellular concentration of homocysteine for the human enzyme. All vitamins except B6 (pyridoxine) were added back individually to levels present in standard medium. Pyridoxine was then added back at a range of concentrations, the “high” dose was 400 ng/mL, the concentration of pyridoxine present in standard medium. Intermediate and low doses of pyridoxine were titrated in starting at 4 ng/mL, a concentration slightly less than that required to achieve maximal growth. Note that even though *S. cerevisiae* requires PLP for growth and makes its own PLP, the level of PLP in wild-type yeast is not sufficient to satisfy the PLP requirement for human CBS when complementing the yeast *cys4* mutation.

For growth-rate measurements, strains containing CBS mutations were pregrown in minimal medium containing GSH to early stationary phase at 30°. Cells were diluted 1:200 in fresh medium lacking GSH, but with varying amounts of pyridoxine (starting cell densities were OD_595_ = 0.01 in a volume of 150 μL). Cell number was measured every 30 min for 72−96 hr in a Tecan GENios plate reader at 27−28° without shaking. For growth-rate calculations, raw OD_595_ values were normalized to an endpoint read after the cells were resuspended. The normalized OD_595_ values were then log_10_ transformed, and the growth rate determined from the slopes calculated between two fixed cell densities (0.05−0.1 for *HEM1* cells, and 0.1−0.2 for *hem1Δ* cells). A plate normalization factor was applied to account for plate-to-plate variations, and the variant growth rates expressed as a ratio relative to the growth rate resulting from the major allele.

### Thermodynamic predictions of the impact of single amino acid variations

The Rosetta computational model for predicting the structural effect of single amino acid substitutions involves a process whereby the side-chain rotamers are sampled while keeping the protein backbone fixed (Bonneau *et al.* 2002; Das and Baker 2008; Kellogg *et al.* 2011; Rohl *et al.* 2004). The calculated energy function consists of an all-atom description including physical and statistical terms. Results from earlier work established a benchmark to assess the predictions from this project. In that work, 985 point mutations in 77 different protein structures were examined. The Pearson correlation coefficient between the predicted and the experimental assessment of function was 0.64 for the entire set (D. Baker and P. Bradley, unpublished data).

### Protein expression and purification

A bacterial expression clone containing a 6His-MBP-CBS fusion was obtained from Macrolab at UC Berkeley (2C-T vector; http://www.addgene.org/29706/). Substitutions within the CBS coding region were then constructed using site-directed mutagenesis and confirmed by DNA sequencing, as was done with the yeast CBS expression constructs. MBP-CBS fusions were expressed in Rosetta 2 (DE3) cells (Novagen; www.emdmillipore.com). Cells were grown in Luria broth with 200 μg/mL ampicillin at 37° to an OD_600_ between 0.6 and 0.8, and then the temperature was shifted to 20° and the cultures were allowed to grow for an additional hour. Protein expression was then induced by the addition of 1 mM IPTG (Gold Biotechnology, St. Louis, MO) and either 0.3 or 0.6 mM δ-ALA (Sigma-Aldrich, St. Louis, MO) and the cells grown at 20° for an additional 15−20 hr.

Cells were harvested by centrifugation and resuspended in 50 mL of lysis buffer (50 mM Tris, pH 8; 300 mM NaCl; 0.5 mM TCEP; 50 μM pyridoxine-HCl; Sigma-Aldrich) with a cocktail of protease inhibitors (Roche Complete, EDTA-free). The cells were then lysed by sonication, and the supernatant fraction collected by centrifugation. Proteins were purified from the lysis supernatant using a Ni-NTA affinity resin (QIAGEN, Valencia, CA), eluted from the column in lysis buffer with 250 mM imidazole, and then dialyzed into lysis buffer to remove the imidazole and concentrate the purified protein. Protein concentrations were determined by measuring the absorbance of the aromatic side chains (Edelhoch 1967).

### Absorbance spectra

Absorbance spectra were taken on purified proteins ranging in concentration from 20 to 100 mM and then normalized to protein concentration for comparison. The ratio of the heme 430 nm absorbance peak and the aromatic 280 nm absorbance peak was one-to-one, indicating nearly 100% heme incorporation in the major allele protein (Majtan *et al.* 2010).

### Proteolysis kinetics

Protease susceptibility of purified proteins was measured using thermolysin. The purified protein sample was diluted to approximately 10 mM in lysis buffer. The reaction was initiated by the addition of thermolysin at a final concentration of 10 μg/mL at room temperature. At defined time intervals a portion of the reaction was removed, and the protease digestion quenched by the addition of ethylenediamine tetraacetic acid at a final concentration of 12.5 mM. Samples were analyzed by 8% sodium dodecyl sulfate polyacrylamide gel electrophoresis.

The MBP-CBS protein fusion, as well as the cleaved CBS and MBP fragments, were excised from the gel and their identity confirmed by mass spectrometry by the following procedure. A nano LC column packed with 10 cm of Polaris c18 5 μm packing material (Varian) was loaded with the protein sample by use of a pressure bomb and washed extensively with buffer A. The column was then directly coupled to an electrospray ionization source mounted on a Thermo-Fisher LTQ XL linear ion trap mass spectrometer. An Agilent 1200 HPLC equipped with a split line, so as to deliver a flow rate of 30 nL/min, was used for chromatography. Peptides were eluted with a 90-min gradient from 100% buffer A to 60% buffer B. Buffer A was 5% acetonitrile/0.02% heptaflurobutyric acid; buffer B was 80% acetonitrile/ 0.02% heptaflurobutyric acid. The programs SEQUEST and DTASELECT were used to identify peptides and proteins from a database consisting of common contaminants and the engineered sequences of the proteins (Eng *et al.* 1994; Tabb *et al.* 2002).

## Results

### CBS-substitution yeast growth phenotypes

We used a cell-based assay to assess the impact of single amino acid substitutions upon the function of the human CBS enzyme. A cDNA clone of the major allele of the human CBS gene, or variant forms of the gene, was expressed in *S. cerevisiae* and tested for functional complementation of a null allele of the orthologous yeast gene, *CYS4*. This strategy has several desirable qualities: (1) expression of the human clone can be driven by a heterologous promoter and terminator so as to minimize transcriptional effects on protein levels; (2) the expression level of the complementing human clone can be modulated so as to restore growth but remain limiting, thereby providing a sensitive assay for both partial loss-of-function alleles or enhanced-function alleles; (3) defined growth media allow for the titration of exogenous pyridoxine or bypass of CBS function entirely by the addition of cysteine in the form of GSH to the growth medium; and (4) the yeast genome can be engineered so as to alter other intermediates of the metabolic pathway. This assay was used to rank order variant activities as well as vitamin, or heme, responsiveness. We recently used this assay to catalog the functional impact of the majority of CBS polymorphisms identified clinically (Mayfield *et al.* 2012).

The focus of this study was to examine the effect of single amino acid substitutions on CBS function and the associated impact of cofactor availability in a more direct fashion. Two strategies were used to construct a new collection of CBS variants, specifically trying to enhance moderate functional impacts. The first was a directed strategy aimed at protein residues not directly involved in either the binding of cofactor or the catalytic pocket, but dispersed throughout the protein, that might result in a range of impacts on protein function. The primary criteria were that the residue be (1) buried within the hydrophobic core (*i.e.*, no solvent accessibility), and (2) distal from the PLP cofactor-binding site. Also, catalytic residues were excluded because mutations of catalytic sites would have clear deleterious effects independent of their effect on the free energy of folding. We used Rosetta-based predictions of the thermodynamic impact of single amino acid substitutions as an approximate guide to be able to test a presumed range of destabilizing mutations. The resulting set of 31 single amino-acid substitutions was predicted to have a range of destabilizing impacts, from mild to severe. The second strategy was random mutagenesis designed to be unbiased with respect to protein residue and substitution and resulted in another set of 27 single-amino-acid substitutions. In total, 58 single amino acid substitutions were constructed in the human CBS gene by these two strategies, and functionally assayed *in vivo*. The complete list of substitutions and their predicted impact on the thermodynamic stability of the protein based on the Rosetta profile is shown in Table 1.

**Table 1:** CBS Protein Variants Constructed and Analyzed

Plasmid (pJR #)	Nucleotide Mutation(s)	Substituted Residue	Rosetta (ΔΔG)	Phenotypic Category	400 ng/mL B6	4 ng/mL B6	2 ng/mL B6	1 ng/mL B6
2985	194, A→T	H65L	1.2	Nonfunctional	0	n/d	n/d	n/d
2993	424, AT→GC	I142A	3.7	Nonfunctional	0	n/d	n/d	n/d
2994	425, T→A	I142N	9.7	Nonfunctional	0	n/d	n/d	n/d
2996	460, CT→GC	L154A	1.8	Nonfunctional	0	n/d	n/d	n/d
2997	461, T→C	L154P	6.7	Nonfunctional	0	n/d	n/d	n/d
2998	529, A→G	K177E	1.2	Nonfunctional	0	n/d	n/d	n/d
3001	566, T→C	V189A	3.1	Nonfunctional	0	n/d	n/d	n/d
3002	620, C→G	A207G	2.0	Nonfunctional	0	n/d	n/d	n/d
3003	629, T→A	L210Q	4.2	Nonfunctional	0	n/d	n/d	n/d
3005	659, T→G	L220R	14.4	Nonfunctional	0	n/d	n/d	n/d
3007	684, C→A	N228K	5.5	Nonfunctional	0	n/d	n/d	n/d
3008	718, AT→GC	I240A	2.1	Nonfunctional	0	n/d	n/d	n/d
3012	755, T→C	V252A	2.6	Nonfunctional	0	n/d	n/d	n/d
3013	772, G→C	G258R	13.7	Nonfunctional	0	n/d	n/d	n/d
3018	829, AT→CC	I277P	5.1	Nonfunctional	0	n/d	n/d	n/d
3023	967, T→G	W323G	6.3	Nonfunctional	0	n/d	n/d	n/d
3035	1153, TTC→CAA	F385Q	5.0	Nonfunctional	0	n/d	n/d	n/d
3038	1268, T→C	L423P	n/a	Nonfunctional	0	n/d	n/d	n/d
3040	1370, G→A	G457E	n/a	Nonfunctional	0	n/d	n/d	n/d
2988	353, T→G	V118G	1.9	Sensitive	0.91 ± 0.04	0.81 ± 0.25	0.49 ± 0.23 *	0.39 ± 0.07 **
2991	379, A→G	I127V	1.5	Sensitive	1.22 ± 0.11	1.25 ± 0.07 *	1.35 ± 0.15	0.97 ± 0.08
2995	424, A→G	I142V	1.3	Sensitive	0.66 ± 0.03 **	0.68 ± 0.07 *	0.53 ± 0.07	0.38 ± 0.10
2999	541, CT→GC	L181A	3.1	Sensitive	0.92 ± 0.23	0.91 ± 0.12	0.56 ± 0.11	0.34 ± 0.22 *
3000	562, A→G	I188V	1.0	Sensitive	1.19 ± 0.23	1.41 ± 0.38	1.42 ± 0.53	1.93 ± 0.64 *
3006	674, A→G	N225S	1.4	Sensitive	0.71 ± 0.10 **	0.62 ± 0.11 **	0.35 ± 0.09 **	0.36 ± 0.07 **
3015	799, A→G	K267E	0.9	Sensitive	0.68 ± 0.38	0.67 ± 0.39	0.47 ± 0.28 **	0.26 ± 0.15 **
3020	856, AT→GC	I286A	1.1	Sensitive	0.52 ± 0.10	0.43 ± 0.05 *	0.18 ± 0.05 *	0.13 ± 0.11 *
3021	877, C→G	L293V	1.1	Sensitive	0.57 ± 0.08	0.50 ± 0.05 *	0.17 ± 0.04 *	0.25 ± 0.06 *
3024	1012, CT→GC	L338A	1.7	Sensitive	0.98 ± 0.11	0.93 ± 0.17	0.77 ± 0.13 *	0.74 ± 0.14
3026	1034, T→C	L345P	−1.2	Sensitive	0.57 ± 0.04 **	0.49 ± 0.07 **	0.16 ± 0.03 **	0.20 ± 0.14 **
3027	1061, T→G	V354G	7.5	Sensitive	0.67 ± 0.09 **	0.72 ± 0.05 **	0.48 ± 0.09 **	0.54 ± 0.10 **
3031	1112, T→C	V371A	2.7	Sensitive	1.15 ± 0.29	1.15 ± 0.37	1.32 ± 0.20 *	1.95 ± 0.54
3036	1153, T→C	F385L	5.5	Sensitive	0.85 ± 0.15	0.72 ± 0.05 **	0.37 ± 0.07 **	0.37 ± 0.03 **
2986	250, A→G	I84V	1.0	Benign	0.99 ± 0.05	1.10 ± 0.28	1.13 ± 0.24	1.31 ± 0.34
2990	370, CT→GC	L124A	2.3	Benign	0.83 ± 0.06	1.00 ± 0.06	0.97 ± 0.07	1.12 ± 0.11
2989	370, C→G	L124V	2.1	Benign	0.76 ± 0.16	1.10 ± 0.18	1.09 ± 0.10	1.62 ± 0.45
2992	418, G→A	D140N	3.0	Benign	1.13 ± 0.28	0.93 ± 0.11	0.86 ± 0.08	0.71 ± 0.07
3004	640, A→G	I214V	1.1	Benign	0.89 ± 0.18	1.16 ± 0.19	1.07 ± 0.17	1.11 ± 0.08
3009	718, A→G	I240V	1.0	Benign	0.90 ± 0.24	1.14 ± 0.21	0.90 ± 0.05	1.02 ± 0.33
3010	721, C→G	L241V	1.4	Benign	1.01 ± 0.13	1.03 ± 0.19	0.91 ± 0.19	0.92 ± 0.41
3011	742, CT→GC	L248A	1.2	Benign	0.81 ± 0.08	0.84 ± 0.11	0.73 ± 0.14	0.71 ± 0.08
3014	791, T→C	I264T	3.3	Benign	1.12 ± 0.33	1.13 ± 0.20	1.05 ± 0.33	1.08 ± 0.31
3016	800, A→T	K267M	−1.8	Benign	1.02 ± 0.18	1.07 ± 0.15	1.10 ± 0.21	1.15 ± 0.18
3017	811, A→G	K271E	0.4	Benign	0.87 ± 0.18	0.89 ± 0.28	0.80 ± 0.20	0.87 ± 0.31
3019	839, T→C	V280A	2.5	Benign	1.10 ± 0.30	1.12 ± 0.27	1.13 ± 0.23	1.41 ± 0.21
3022	931, A→G	I311V	0.7	Benign	1.03 ± 0.15	1.33 ± 0.50	1.12 ± 0.44	1.41 ± 0.75
3025	1023, A→T	Q341H	−2.5	Benign	1.09 ± 0.23	0.85 ± 0.20	0.86 ± 0.19	0.86 ± 0.10
3028	1067, T→C	V356A	1.3	Benign	0.98 ± 0.12	1.04 ± 0.06	0.94 ± 0.00	0.92 ± 0.08
3029	1070, C→G	A357G	1.6	Benign	1.09 ± 0.16	1.17 ± 0.23	0.99 ± 0.23	0.77 ± 0.19
3030	1073, T→C	V358A	1.6	Benign	0.92 ± 0.10	0.90 ± 0.05	0.87 ± 0.14	0.73 ± 0.08
3032	1115, T→C	V372A	2.7	Benign	0.75 ± 0.39	0.85 ± 0.48	0.68 ± 0.38	0.68 ± 0.37
3033	1120, CT→GC	L374A	2.3	Benign	1.09 ± 0.10	1.06 ± 0.20	1.17 ± 0.24	1.28 ± 0.22
3034	1147, A→T	T383S	1.1	Benign	0.95 ± 0.15	0.87 ± 0.14	0.70 ± 0.10	0.83 ± 0.22
3037	1223, G→T	W408L	n/a	Benign	1.04 ± 0.03	1.13 ± 0.17	1.19 ± 0.16	1.08 ± 0.10
3039	1298, A→T	H433L	n/a	Benign	0.98 ± 0.22	0.93 ± 0.30	0.83 ± 0.21	0.72 ± 0.13
3041	1468, A→C	I490L	n/a	Benign	0.86 ± 0.49	0.74 ± 0.42	0.74 ± 0.43	0.87 ± 0.49
3042	1646, A→G	D549G	n/a	Benign	0.95 ± 0.13	0.80 ± 0.11	0.69 ± 0.10	0.65 ± 0.12
3043	1675, T→C	Y559H	n/a	Benign	1.36 ± 0.10	1.18 ± 0.32	1.15 ± 0.35	1.07 ± 0.37

The 58 single amino-acid substitutions constructed within the human CBS coding region are listed. The engineered nucleotide change and the resulting change in amino acid residue are shown (reference sequence NM_000071). The calculated change in free energy based on the Rosetta prediction algorithm for each substitution is also listed (ΔΔG, kcal/mol), with the exception of seven substitutions located in the N-terminal portion of the protein that is not part of the structure and therefore no predictions were available (indicated as n/a). All of the protein variants were assigned to one of three phenotypic categories based on empirical measurements of growth: benign, sensitive, or nonfunctional. The relative growth rates of yeast containing 39 single-amino-acid human CBS substitutions with some degree of function are listed. Growth rates are expressed as a ratio relative to the yeast strain expressing the major human CBS allele, grown with the same amount of exogenous pyridoxine supplementation (either 400, 4, 2, or 1 ng/mL pyridoxine), with the SD indicated (determined from at least three independent yeast transformants). Growth rates that differed significantly from the major allele in the same media conditions are indicated either by a single asterisk (*P* < 0.01) or a double asterisk (*P* < 0.001). Growth rates that were not determined are indicated (n/d). CBS, cystathionine β-synthase.

The observed growth phenotypes were assigned to one of three categories. The first comprised those changes that failed to confer growth even at the highest concentration of exogenous pyridoxine supplementation (400 ng/mL). These 19 substitutions created a nonfunctional enzyme as defined by the *in vivo* growth assay. The remaining two categories together comprised 39 substitutions that were able to confer growth to some degree. Their *in vivo* activities were determined at four concentrations of exogenous pyridoxine supplementation (Table 1). Twenty-five of these substitutions supported growth rates comparable to wild type at all four concentrations of pyridoxine supplementation, and were defined as benign. The final category of 14 substitutions conferred a growth rate that was significantly different from that of yeast with the major allele of human CBS in at least one concentration of pyridoxine supplementation (pair-wise *t* test; *P* < .01). Although the majority of these substitutions exhibited slower growth rates relative to wild type, particularly at low pyridoxine concentrations, three substitutions (I127V, I188V, and V371A) conferred better growth than wild type. We refer to this category of 14 substitutions as being cofactor sensitive.

### The distributed nature of functional classes of mutations

Some alleles led to proteins with decreased yet remediable function. For these mutants, their loss or reduction in function could be compensated by the binding of a cofactor. If the effect of these mutations on the enzyme were local to the altered site, remediable alleles would be expected to be close in structure to the cofactor-binding site. If, however, the effect were more global, such as a general destabilization of the protein, then remediable alleles may occur at a considerable distance from the cofactor-binding site.

**Figure 1:**
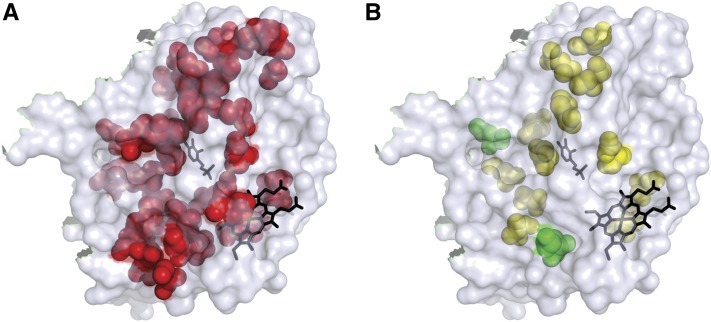
Positions of the altered CBS amino acid residues on the 3D structure of the truncated CBS protein. The pyridoxine and heme cofactors are shown in black. (A) Forty-four amino acid residues representing 51 of the 58 substitutions are shown in red. Seven of the substitutions were in residues not included in the 3D structure and are not shown. (B) The fourteen residues that, when substituted, resulted in CBS protein variants with altered cofactor sensitivity *in vivo*. All variants demonstrated altered sensitivity to pyridoxine; the two variants that had altered sensitivity to both heme and pyridoxine (K267E and L345P) are shown in green.

We used the structure of a truncated version of the major allele protein as a framework for exploring the relative distribution of different classes of variants. We mapped the positions of the 44 mutated residues with positional information (representing 51 mutations, 7 mutations were in residues not included in the solved structure) to examine if there were any “hot spots” for the phenotypic categories (Figure 1). There was no obvious clustering of any phenotypic classes to any particular regions. Indeed, the mutated residues were located throughout the structure and the different phenotypic categories were interspersed. The lack of geographic bias added strength to the view that the phenotypic categories were the result of distributed influences and were not limited to region-specific properties.

To explore possible associations between physical characteristics of substitutions and observed growth phenotype in a more quantitative fashion, alleles were grouped based their growth phenotype: nonfunctional, sensitive, or benign; each of these three groups were further divided based on the method of molecular construction, directed or random (six groups in all). For each category, five physical characteristics were determined (distance between the substitution and heme, distance between the substitution and PLP, solvent accessibility, regular secondary structure, and crystal structure B-factor), and category averages were calculated. For comparison, an equal number of alleles were randomly assigned to each category 10,000 times and category averages for the same five measurements performed allowing a rigorous statistical test for the probability that the test category is higher or lower than random.

As expected, the directed variants had significantly less solvent accessibility than the randomized controls because this was a criterion in their selection. This correlation provided a positive control that the analysis could detect correlations if they existed among the data. Among the randomly generated variants, those with nonfunctional or sensitive phenotypes were also less solvent accessible than either the controls or the benign random substitutions. However, no other physical parameter examined was characteristic of any of the phenotypic categories. The remediable alleles were no closer to either of the cofactor binding sites, nor did they have altered occurrence in regular secondary structure, or correlate with high or low crystallographic B-factor values.

### Correlations of thermodynamic predictions with growth phenotypes

**Figure 2:**
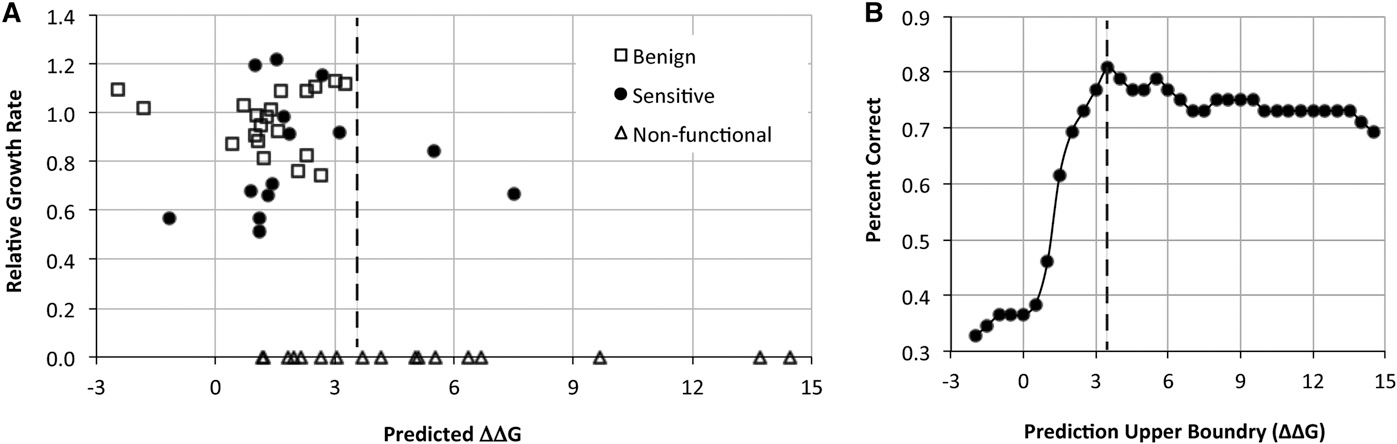
Yeast *in vivo* growth complementation assay for human CBS function. (A) Scatter plot showing the predicted change in free energy (Predicted ΔΔG, x-axis) for 52 human single-amino-acid CBS substitutions *vs.* their conferred growth rates expressed relative to that of the major human CBS allele grown with 400 ng/mL pyridoxine supplementation in *HEM1* yeast strains (y-axis). The alleles were categorized by growth phenotype as benign, sensitive, or nonfunctional (see *Results*). (B) Assessment of the calculated changes in free energy to predict the phenotypic outcome for 52 human CBS substitutions. The alleles were binned based on the predicted change in free energy (ΔΔG) in 0.5 kcal/mol increments. For each incremental threshold a percentage “correct” was calculated by summing the number of alleles growing below the threshold added to the number of alleles not growing above the threshold, divided by the total number of alleles (*i.e.*, 52). The dotted line indicates a predicted ΔΔG of 3.5 kcal/mol Rosetta units.

Although the Rosetta algorithm was used to select mutations with a range of predicted effects on stability, the correlation between the predicted effects on stability and observed effects on growth was poor. The only reliable correlations were for those variants predicted to be severely destabilizing (>4 kcal/mol on the Rosetta scale). Nine of the 11 substitutions in this category resulted in a no-growth, no-remediation phenotype (Figure 2A). The two remaining substitutions (F385L and V354G) both were impaired and sensitive to low levels of pyridoxine, in close agreement with a previous study (Wei *et al.* 2010). Thus the 3.5- to 4-kcal/mole ΔΔG prediction marked a threshold above which all examined CBS variants were impaired, and indeed most were completely nonfunctional (Figure 2B).

In contrast, the majority of mutations were predicted to cause a more modest ΔΔG effect and were selected precisely because of their predicted intermediate effects on stability. These variants fell into all three growth-phenotype categories (Figure 2A). There were no significant correlations between growth phenotype and the predicted change in stability for mutations in this range of destabilization.

### Characterization of cofactor remediation

Cofactor-remediable substitutions occurred at a significant distance from the binding site of that cofactor, which is consistent with the hypothesis that the effects of remediable alleles were not local, and were more likely a result of a modest destabilization of the protein. If true, the cofactor sensitivity of a given variant may not be specific to one cofactor. That is, interaction with a different cofactor, which also binds at a distance from the substitution, may also provide enough stabilization to at least partially compensate for the stability lost due to the substitution. We examined this question by transforming the first nine B6-remediable alleles that we identified into a yeast *hem1Δ* strain in which we could control the relative level of intracellular heme, the other cofactor for CBS, by supplementing with either high or low concentrations of δ-amino levulinic acid (δ-ALA). δ-ALA is the product of Hem1p and a precursor in heme biosynthesis (Table 2). Two alleles, K267E and L345P, which were sensitive to low pyridoxine levels, were also extremely sensitive to low δ-ALA concentrations. Neither of these substitutions was particularly close to either cofactor-binding site; in fact L345P is on the opposite side of the monomer from the heme-binding site (Figure 1). Therefore, these two alleles encoded proteins that were severely impaired by low concentrations of either cofactor, yet could be significantly remediated by high concentrations of either.

**Table 2:** Growth Rates Supported by CBS Variants in a *hem1Δ* Background

Substituted Residue	400 ng/mL B6 and 50 µg/mL δ-ALA	400 ng/mL B6 and 5 µg/ml δ-ALA	2 ng/mL B6 and 50 µg/ml δ-ALA	2 ng/mL B6 and 5 µg/mL δ-ALA
V118G	0.90 ± 0.06	0.78 ± 0.12	0.60 ± 0.15	0.56 ± 0.07
I142V	0.82 ± 0.07	0.74 ± 0.12	0.69 ± 0.14	0.87 ± 0.08
L181A	0.64 ± 0.06	0.59 ± 0.03	0.57 ± 0.09	0.59 ± 0.08
N225S	0.74 ± 0.11	0.66 ± 0.06	0.55 ± 0.12	0.45 ± 0.07
K267E	0.61 ± 0.00	n/d	0.57 ± 0.18	n/d
I286A	0.64 ± 0.03	0.58 ± 0.02	0.46 ± 0.06	0.34 ± 0.05
L293V	0.69 ± 0.07	0.67 ± 0.10	0.47 ± 0.07	0.36 ± 0.06
L345P	0.58 ± 0.01	0.07 ± 0.03	0.25 ± 0.06	0.05 ± 0.01
F385L	0.77 ± 0.05	0.69 ± 0.07	0.58 ± 0.10	0.54 ± 0.11

Relative growth rates of a subset of 9 B6-remediable single amino-acid human CBS substitutions expressed in yeast are listed. Each variant was grown in four media conditions; 400 ng/mL pyridoxine and 50 µg/mL ∂-ALA, 400 ng/mL pyridoxine and 5 µg/mL ∂-ALA, 2 ng/mL pyridoxine and 50 µg/mL ∂-ALA, and 2 ng/mL pyridoxine and 5 µg/mL ∂-ALA. As in Table 1, growth rates are expressed as a percentage relative to the yeast strain expressing the major allele of human CBS grown with the same media. Averages were determined from at least three independent yeast transformants of each CBS variant. Conditions in which a variant was unable to grow sufficiently to determine a growth rate are indicated (n/d). CBS, cystathionine β-synthase.

### Physical analysis of heme incorporation

**Figure 3:**
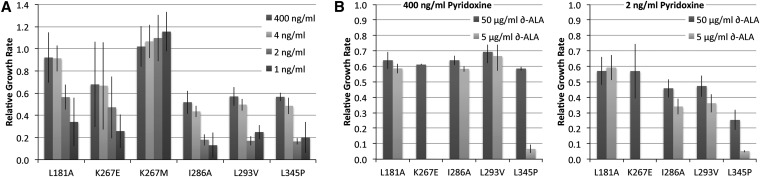
CBS variant growth rates at various levels of exogenous cofactor supplementation. (A) Growth rates for six variants grown with four levels of pyridoxine supplementation (400, 4, 2, and 1 ng/mL) in *HEM1* yeast strains. Growth rates are expressed relative to yeast with the major human CBS allele grown with the same media conditions. (B) Growth rates for five of the same variants grown in *hem1Δ* strains, in either a high level of pyridoxine (400 ng/mL, left panel) or a low level of pyridoxine (2 ng/mL, right). Additionally, these strains were grown with either high δ-ALA supplementation (50 μg/mL) or low δ-ALA supplementation (5 μg/mL). The K267M variant was omitted because it did not show any sensitivity to pyridoxine levels (A). All error bars indicate the standard deviation of at least three independent transformants.

To probe the nature of the remediable alleles, we focused on the cofactor sensitivity of a subset of substitutions for *in vitro* analysis. Considering that substitutions predicted to have changes in free energy less than 3.5 kcal/mol produced protein variants of all phenotypic categories, we selected six variants for this analysis that were predicted to have modest destabilizing effects, yet represented three of the phenotypic categories (the nonfunctional alleles were omitted). Of these, two variants exhibited heightened sensitivities to levels of both cofactors, pyridoxine and heme (K267E and L345P), an additional three variants showed sensitivity to reduced pyridoxine levels but not heme levels (L181A, I286A, and L293V), and the final variant selected was benign (K267M; Figure 3, A and B).

**Figure 4:**
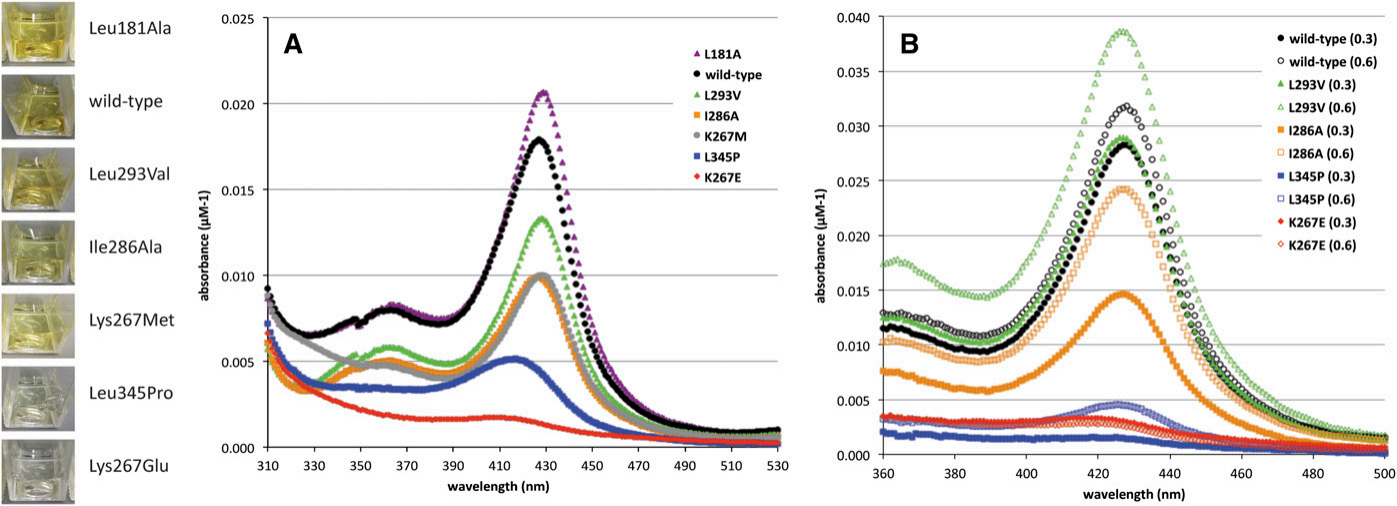
Heme content of purified variant CBS proteins. Photographs of cuvettes containing equal concentrations of each purified protein are shown on the left. (A) Absorption spectra demonstrating the level of heme incorporation for seven CBS proteins. Bacterial cells expressing the various CBS proteins were supplemented with 0.3 mM δ-ALA in the growth medium. (B) Increased incorporation of heme for the CBS variants when expressed with an increased concentration of exogenous δ-ALA in the growth medium (0.3 mM [solid symbols] *vs.* 0.6 mM [open symbols]).

For the *in vitro* studies, the full-length CBS coding region was fused to that of the maltose binding protein (MBP), which facilitated the solubility of bacterially expressed CBS variants. To aid in the intracellular production of heme and its subsequent incorporation into the CBS protein during expression, the bacterial growth medium was supplemented with δ-ALA (Kery *et al.* 1994). This MBP-CBS fusion protein was soluble when expressed in bacteria and could be readily purified and assayed. The purification yield varied significantly among the seven selected proteins (major allele and six variants) and correlated with the intensity of a yellow/brown color of the purified protein (Figure 4). This observation suggested that the amount of heme associated with the purified variants also varied (Kery *et al.* 1994). Indeed, the amount of protein-bound heme, measured spectrophotometrically, varied significantly among the protein variants and correlated with the observed color (Figure 4A) and with activity *in vivo* as measured by growth rate.

The observed differences in heme sensitivity *in vivo* and heme incorporation *in vitro* suggested both that this collection of proteins varied in their ability to bind heme, and that changes in the availability of heme influenced its incorporation, and subsequent enzymatic function. For example, K267E and L345P, which were most sensitive to heme availability *in vivo*, incorporated the least heme, as measured *in vitro*. Several of these variants, including the major allele, were expressed in cells grown at two concentrations of δ-ALA, purified and evaluated for heme incorporation (Figure 4B). Increased heme availability resulted in marginal gains in the low level of heme incorporated into L345P and no detectable increase in K267E, consistent with the extreme sensitivity of these two variants to lowered levels of heme. In contrast, L293V, I286A and the major allele form of CBS had more associated heme under these conditions, presumably reflecting a greater fraction of CBS molecules that had incorporated heme, and supported greater growth.

### Physical analysis of protein stability

One method to interrogate the stability and dynamics of a purified protein is to examine its sensitivity to proteases (Park and Marqusee 2006). The differences in *in vivo* function, heme content in the purified proteins, and their altered sensitivity to pyridoxine levels collectively implied that the alleles described in Figure 4 likely resulted in a conformational perturbation of the protein. Therefore, we examined their relative sensitivity to thermolysin cleavage. The thermolysin-sensitivity of the major allele full-length CBS and variants has been used previously to detect whether substitutions affected the conformational stability for other variants of CBS (Hnizda *et al.* 2012a).

**Figure 5:**
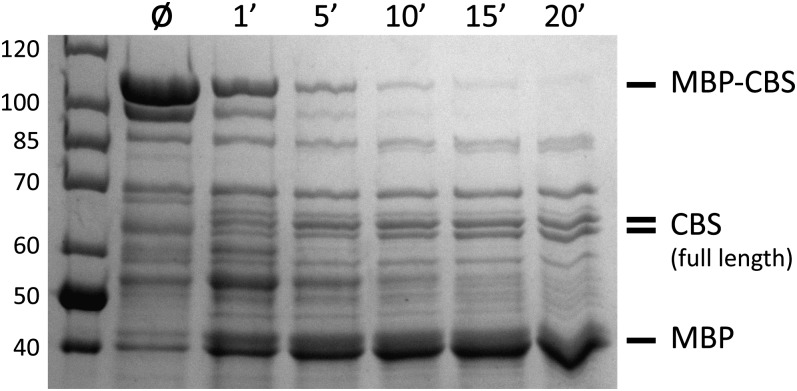
Protease digestion of the purified major allele MBP-CBS fusion protein. After treatment with thermolysin for the indicated time (minutes), the digested protein is separated by polyacrylamide gel electrophoresis. The full-length CBS protein (with MBP removed) runs as a doublet; the identities of all of the labeled bands were confirmed by Mass-Spectrometry analysis. Iota marks the lane with no protease.

**Figure 6:**
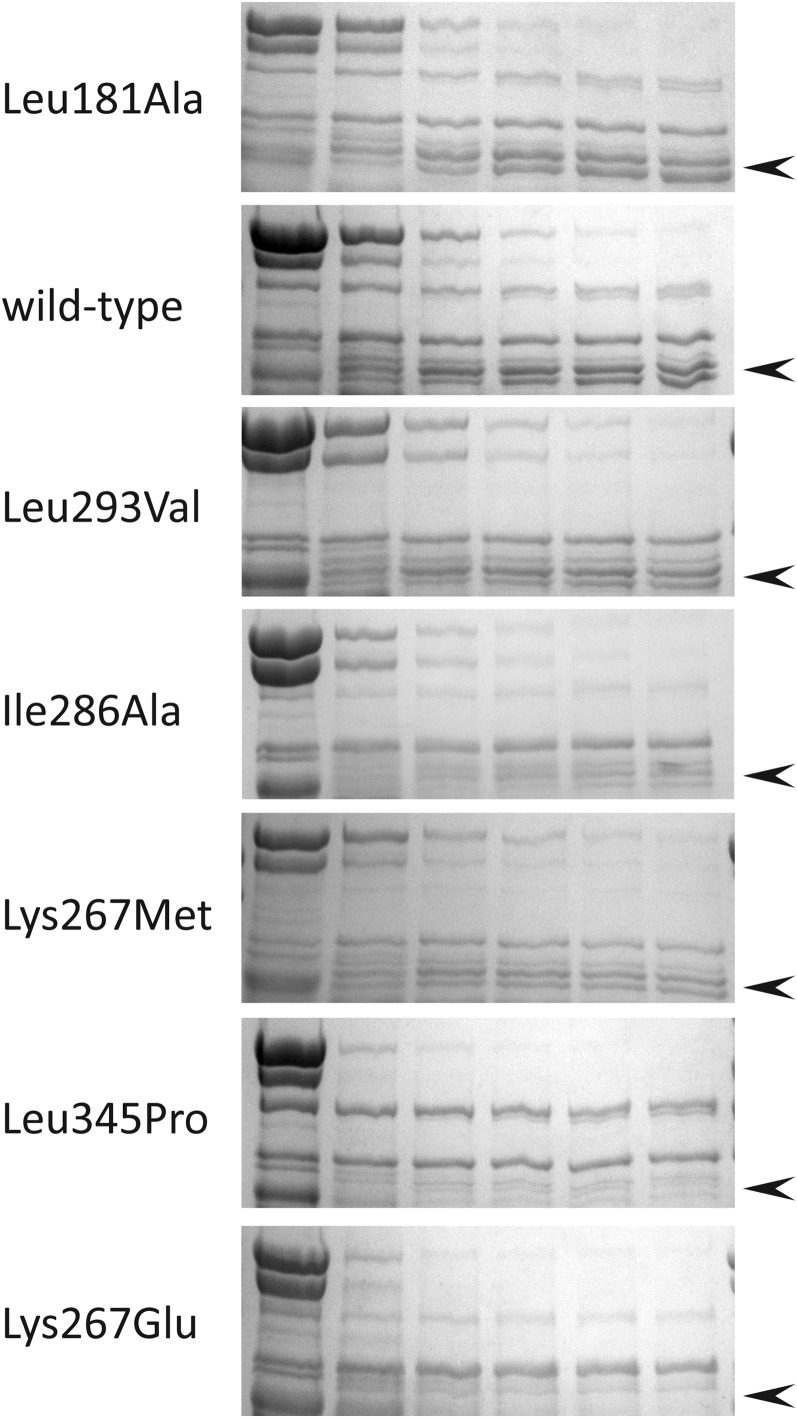
Protease digestion of seven purified MBP-CBS protein alleles. The conditions of the thermolysin digests are as in Figure 5, and the order of proteins from the top is that of heme incorporation, from highest to lowest. In each case the relevant doublet band of full-length CBS is indicated by the arrow head. The lower portion of the gel showing the cleaved MBP band is not shown.

Under the conditions used here, the MBP fragment was quickly cleaved from the full-length CBS domain, even at the lowest protease levels tested. The remaining full-length major-allele CBS remained largely resistant to further degradation by thermolysin treatment for 20 min (Figure 5). Longer protease time points eventually degraded the full-length CBS (not shown). The sensitivity to thermolysin digestion varied significantly among the alleles. Importantly, the degree of sensitivity correlated with heme content. The less heme incorporated in the protein, the more sensitive the CBS variant was to protease digestion (Figure 6). These results implied that heme-binding stabilized the protein to proteolysis.

## Discussion

In this study we interrogated the relationship between protein structure and cofactor-level sensitivity of mutant variants of human CBS, a B6-dependent enzyme with a key role in metabolism. Importantly, cofactor-remediable variants occurred at positions dispersed throughout the structure of the protein. The dually remediable variants, responsive to both B6 and heme cofactors, were striking in that the changes in these variants were not proximal to the binding site of either cofactor. The dual B6- and heme-level sensitivities of two variants in particular raised the possibility that iron deficiency, or other contributions to heme function, might also impact enzyme function in humans carrying these alleles.

Five of the six single amino acid substitutions of CBS selected for physical analysis exhibited altered sensitivity to the B6 cofactor *in vivo* (the sixth, functionally benign K267M variant, was included in this set as a control), but displayed differing phenotypes with respect to heme-level sensitivity. Three variants, (L181A, I286A, and L293V) exhibited sensitivity to reduced pyridoxine levels relative to the major allele, but no heightened sensitivity to reduced heme levels based upon growth-rate measurements. However, there was a range of heme incorporation among these alleles *in vitro*. The L181A variant appeared to incorporate a level of heme similar to the protein encoded by the major allele, although the other two CBS variants incorporated less heme. These variants established that heightened B6-level sensitivity was not necessarily coupled with discernable effects on heme loading or conformational stability, and suggested that there may be a threshold with respect to heme loading and protein function *in vivo*.

Perhaps the most interesting alleles were those that encode K267E and L345P, which exhibited the most extreme *in vivo* sensitivity to reduced levels of either pyridoxine or heme. Upon purification both variant proteins bound far less heme than wild-type CBS, and exhibited the most enhanced thermolysin sensitivity of the seven CBS versions tested. Heme incorporation into CBS protein appears to be obligatorily co-translational, and reduced heme binding in turn affects PLP binding (Kery *et al.* 1994; Oliveriusova *et al.* 2002). Thus variants with reduced heme binding would be expected to alter PLP binding and yield a protein with reduced function, as was observed for these two alleles. But at a fixed level of heme availability, and presumably therefore at a fixed level of heme incorporation, these two variants also showed altered activity to pyridoxine concentrations *in vivo*. It would appear that variants with heightened sensitivity to the levels of two cofactors commonly have heightened conformational flexibility, as measured by protease sensitivity, relative to variants with more specific sensitivities.

Taken to a logical conclusion, these striking examples raise the possibility that a small molecule ligand that binds to one position of a protein can suppress the impact of a destabilizing mutation at a distant site. This notion is consistent with the observation that the physical impacts of a number of clinical CBS alleles result in subtle alterations of protein structure (Hnizda *et al.* 2012b) and that small molecule chaperones can rescue function (Kopecka *et al.* 2011; Majtan *et al.* 2010). If so, small molecules that restore function to variant proteins could have significant translational importance. Moreover, there is growing recognition of cryptic binding sites for small molecules that are obscured in crystal structures but revealed in molecular dynamic simulations (Bowman and Geissler 2012).

Clearly, experimental evaluation of all amino acid substitutions in the human proteome would be technically daunting. Hence robust computational prediction of the impact of variation would be desirable if personalized medicine is to reach its full potential. However, the results described here demonstrate that single amino acid substitutions that are calculated to have a modest impact on protein stability can nevertheless impact protein function, ranging from nonfunctional to benign. The needed improvements in predictive methods rely on datasets that interrogate the impact of many alleles of a protein. The 58 alleles of CBS described here, combined with previous studies, offer an increasingly useful resource for such developments.
